# Motor neurons and the generation of spinal motor neuron diversity

**DOI:** 10.3389/fncel.2014.00293

**Published:** 2014-10-09

**Authors:** Nicolas Stifani

**Affiliations:** Medical Neuroscience, Dalhousie UniversityHalifax, NS, Canada

**Keywords:** motor neurons, development, central nervous system, spinal cord, transcription factors, spinal motor neuron, lower motor neuron

## Abstract

Motor neurons (MNs) are neuronal cells located in the central nervous system (CNS) controlling a variety of downstream targets. This function infers the existence of MN subtypes matching the identity of the targets they innervate. To illustrate the mechanism involved in the generation of cellular diversity and the acquisition of specific identity, this review will focus on spinal MNs (SpMNs) that have been the core of significant work and discoveries during the last decades. SpMNs are responsible for the contraction of effector muscles in the periphery. Humans possess more than 500 different skeletal muscles capable to work in a precise time and space coordination to generate complex movements such as walking or grasping. To ensure such refined coordination, SpMNs must retain the identity of the muscle they innervate. Within the last two decades, scientists around the world have produced considerable efforts to elucidate several critical steps of SpMNs differentiation. During development, SpMNs emerge from dividing progenitor cells located in the medial portion of the ventral neural tube. MN identities are established by patterning cues working in cooperation with intrinsic sets of transcription factors. As the embryo develop, MNs further differentiate in a stepwise manner to form compact anatomical groups termed pools connecting to a unique muscle target. MN pools are not homogeneous and comprise subtypes according to the muscle fibers they innervate. This article aims to provide a global view of MN classification as well as an up-to-date review of the molecular mechanisms involved in the generation of SpMN diversity. Remaining conundrums will be discussed since a complete understanding of those mechanisms constitutes the foundation required for the elaboration of prospective MN regeneration therapies.

## Introduction

Motor neurons (MNs) are neuronal cells located in the central nervous system (CNS) controlling a variety of downstream targets. There are two main types of MNs, (i) upper MNs that originate from the cerebral cortex and (ii) lower MNs that are located in the brainstem and spinal cord. Among the latest, spinal MNs (SpMNs) have been intensively studied during the last decades and therefore provide an interesting framework for further molecular characterization. SpMNs are located in the ventral horn of the spinal cord and control effector muscles in the periphery. They form the ultimate and irreplaceable component of the neuronal circuitry since there is no alternative route to convey the commands from the processing centers located in the CNS to the effector muscles in the periphery. Their axon extending through several meters in mammals constitute an exceptional and unique anatomical feature. SpMNs are therefore the longest known cell type.

Complex movements such as walking or grasping require the cooperation of several dozens of muscles. Additionally, sensory-motor feedback loops are essential for the real-time tuning of gestures. To ensure such refined coordination, SpMNs must acquire and retain the identity of muscles they innervate as well as be integrated in a coherent and functional neuronal circuitry. Hollyday et al. (1977) and Landmesser (1978) initially described the anatomical organization of SpMNs with respect to their muscle targets. Authors acknowledged an association between SpMNs' positions and their respective muscle target in the periphery. Ultimately these findings led to the concept of MN pool, which is defined as a compact anatomical group of MNs sharing similar intrinsic characteristics and connecting to a single target in the periphery. Because of their unique and irreplaceable function, diseases that involve loss of MNs such as progressive muscular atrophy, spinal muscular atrophy, primary lateral sclerosis, and amyotrophic lateral sclerosis, are rapidly debilitating, as only symptomatic treatments are available. Understanding the molecular mechanisms underlying SpMN diversity is among the fundamental steps required to elaborate successful regenerative therapies in the future. Here, we provide a complete description of MN classification to then review in depth the organization as well as the molecular mechanisms involved in the generation of SpMNs.

## Motor neuron classification

MNs are exceptional cell types that can be divided into two main categories according to the location of their cell body: (i) upper and (ii) lower MNs. Upper and lower MNs must be considered as distinct entities despite of their shared nomenclature. Table 1 summarizes the differences between the two in terms of cell body location, neurotransmitter, targeting, and symptoms upon lesion and emphasizes the inappropriateness of a similar appellation to name both entities.

**Table 1:** **Comparison between upper and lower MNs**.

	**Upper MNs**	**Lower MNs**
Location	Cortex	Brainstem and SC
Neurotransmitter	Glutamate	Acetylcholine
Targeting	Within the CNS	Outside the CNS
Symptoms upon lesion	Spasticity	Paralysis

Upper and lower MNs diverge in their cell body location, neurotransmitter, targeting and symptoms upon lesion.

### Upper motor neurons

Upper MN cell bodies are located in the pre-motor and primary motor region of the cerebral cortex also known as the “motor strip.” Since upper MNs make glutamatergic connections with lower MNs located in the CNS, they are exclusively confined to the latter. Typical clinical symptoms of upper MN lesion include uncontrolled movement, decreased sensitivity to superficial reflex stimulation and spasticity (Ivanhoe and Reistetter, 2004). The organization of upper MNs is complex and can't be completely and accurately described in this review that primarily focuses on molecular mechanisms that generate SpMN diversity. Readers are invited to refer to the chapter 16 entitled “Upper Motor Neuron Control of the Brainstem and Spinal Cord” from Purves and Williams (2004) for more information.

### Lower motor neurons

Lower MN cell bodies are located in specific nuclei in the brainstem as well as in the ventral horn of the spinal cord and therefore, alike upper MNs, are settling within the CNS. The remarkable characteristic of lower MNs is their axonal extension and connection outside of the CNS. Lower MNs are cholinergic and receive inputs from upper MNs, sensory neurons (SNs) as well as from interneurons (INs). Paralysis is a typical clinical symptom of lower MN lesions since once damaged there is no alternative route to convey the information to the muscle targets in the periphery. Lower MNs are classified into three groups according to the type of target they innervate: (i) branchial, (ii) visceral, and (iii) somatic MNs.

#### Branchial motor neurons

Branchial MNs are located in the brainstem and form, together with SNs, the cranial nuclei. They innervate branchial arch derived muscles of the face and neck through 5 cranial nuclei: the trigeminal (V), facial (VII), glossopharyngeal (IX), vagus (X) and accessory (XI) nerves. Despite their similar function, muscles of the neck and the face differ from other skeletal muscles in their embryological origin since they do not derive from the somites, but instead from the branchial arches. Such developmental difference is mirrored by specific characteristics reviewed in depth by Chandrasekhar (2004).

#### Visceral motor neurons

Visceral MNs belong to the autonomic nervous system (ANS) responsible for the control of smooth muscles (i.e., heart and arteries) and glands. The ANS can be described as the association of two components: (i) preganglionic MNs located in the CNS connected to ganglionic neurons belonging to the peripheral nervous system (PNS). In turn, peripheral ganglionic neurons target to the final effector organ. Additionally, the ANS is anatomically and functionally divided into two structures: (i) the sympathetic system and (ii) the parasympathetic system.

***Motor neurons of the sympathetic system***. The sympathetic nervous system is involved in the traditional “fight or flight” responses, recruiting energy storage, increasing awareness, and leading to a global activation of the body metabolism. Central MNs of the sympathetic system are located in the spinal cord from the thoracic segment 1 (T1) to the lumbar segment 2 (L2). These MNs have an intermedio-lateral position and constitute the preganglionic column (PGC) that will be described below. They connect to 3 different targets: two chains of ganglia adjacent to the spinal cord named (i) paravertebral and (ii) prevertebral as well as directly to (iii) the chromaffin cells of the adrenal medulla responsible for the release of the catecholamines (i.e., adrenaline and noradrenaline) in the circulation, in response to stress stimuli. On the other hand, paravertebral and prevertebral ganglia connect to a wide variety of targets including the heart, lungs, kidneys, intestines and the colon.

***Motor neurons of the parasympathetic system***. The parasympathetic system controls glands secretion and activates the gastrointestinal tract as well as sexual behavior, which are summarized as “rest and digest” functions. Central MNs of the parasympathetic system are located in the brainstem and contribute to the formation of the cranial nerves (III, VII, IX, and X). Parasympathetic MNs are also found in sacral segments 2 to 4 (S2–S4) of the spinal cord. They innervate ganglia located in the proximity of the peripheral targets such as the heart, bladder, lungs, kidneys, and pancreas.

In summary, visceral central MNs from the sympathetic and parasympathetic systems relay information from the CNS to ganglionic neurons of the PNS. In turn those ganglia antagonistically control a large number of various visceral targets. In contrast to branchial mentioned previously and somatic MNs described below, visceral MNs do not directly connect to the final effector. As a result, they constitute an anatomical and functional exception among lower MNs.

#### Somatic motor neurons

**Figure 1:**
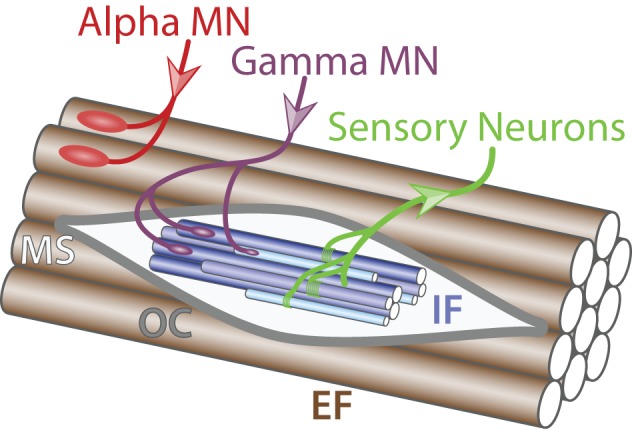
**Muscle innervation**. Schematic of muscle fibers on the longitudinal section (adapted from Purves and Williams, 2004). Alpha MN (red) innervates (incoming arrow) extrafusal muscle fibers (EF, brown) whereas gamma MN (purple) connects to intrafusal fibers (IF, blue) within the muscle spindle (MS, light gray) surrounded by the outer capsule (OC, dark gray). Sensory neurons (green) carry information from the intrafusal fibers to the central nervous system (outgoing arrow).

Somatic MNs are located in the Rexed lamina IX in the brainstem and the spinal cord and innervate skeletal muscles responsible for movements (Rexed, 1954). MNs form coherent groups connecting to a unique muscle target defined as MN pools. Somatic MNs can be divided into 3 groups: (i) alpha, (ii) beta, and (iii) gamma according to the muscle fiber type they innervate to within a specific muscle target (Figure 1). A motor unit defines a single MN together with all the muscle fibers it innervates. Interestingly, motor units are homogeneous: a MN innervates muscle fibers of a single type. This observation suggests selectivity in the establishment of neuromuscular connectivity and/or a coordinated maturation between a MN and its targeted fibers. Intuitively, the diversity of MNs mirrors the diversity of targets they innervate. Therefore, to better describe somatic MN diversity, a brief description of skeletal muscle physiology will be provided.

Three classes of muscles can be anatomically and functionally distinguished: (i) cardiac muscles, (ii) smooth muscles and (iii) skeletal muscles. Cardiac muscles are responsible for the rhythmic contraction of the heart while smooth muscles control the diameter of blood vessels and the internal digestive and secretion organs. Both smooth and cardiac muscles are innervated by the ANS (described above). In contrast, somatic MNs exclusively innervate skeletal muscles that are the most abundant muscle class, with around 639 different muscles in the human body (Stone and Stone, 2009). Skeletal muscles are firmly attached to the skeleton by the tendons and are responsible for both posture and movement. Developmentally, skeletal muscles derive from the paraxial mesoderm that produces the somites, which in turn generate muscle precursor cells called myoblasts. Those cells migrate toward the periphery and fuse to form the body of the muscle. Physiologically, skeletal muscles are composed of 2 structures: (i) extrafusal fibers, generating the force and (ii) muscle spindles providing proprioceptive information on the position and extension status of the muscle. Muscle spindles are composed of several intrafusal fibers enveloped by a collagen sheath named the outer capsule. There are three kinds of intrafusal fibers with specific characteristics: (i) dynamic nuclear bag fibers (B1), (ii) static nuclear bag fibers (B2 fibers) and (iii) nuclear chain fibers. Analogously, extrafusal fibers are divided into 3 types according to their physiological and molecular properties: (i) slow-twitch fatigue-resistant (SFR), (ii) fast-twitch fatigue-resistant (FFR) and (iii) fast-twitch fatigable (FF). Table 2 summarizes the principal characteristics of the three extrafusal muscle fibers.

**Table 2:** **Characteristics of extrafusal muscle fiber types**.

		**SFR**	**FFR**	**FF**
Anatomical properties	Fiber Diameter	Small	Intermediate	Large
	Color	Red	Light red	White
	Capillaries	Many	Many	Few
	Myoglobin level	High	High	Low
	Mitochondria	Many	Many	Few
Physical properties	Duration use	Hours	Minutes	< Minute
	Power produce	Low	High	Very high
	Recruitment order	First	Second	Third
	Fatigue sensitivity	Slow	Intermediate	Fast
	Contraction velocity	Slow	Fast	Fast
	Function activity	Posture	Normal movements	Intense movements
Metabolic properties	Myosin ATPase activity	Slow	Fast	Fast
	ATP synthesis	Aerobic	Intermediate	Anaerobic
	Glycogen stores	Low	Intermediate	High
	Oxidative capacity	High	Intermediate	Low
	Glycolytic capacity	Low	Intermediate	High
	Energy storage	Triglycerides	Creatine Phosphate	Creatine Phosphate
			Glycogen	Glycogen

Slow-twitch fatigue-resistant (SFR), Fast-twitch fatigue-resistant (FFR), and Fast-twitch fatigable (FF) fibers differ in term of anatomical, physical, and metabolic properties.

Mirroring the diversity of both intra- and extrafusal fiber types in a muscle, somatic MNs are further sub-divided into 3 types: (i) alpha, (ii) beta and (iii) gamma that will be further described below.

**Figure 2:**
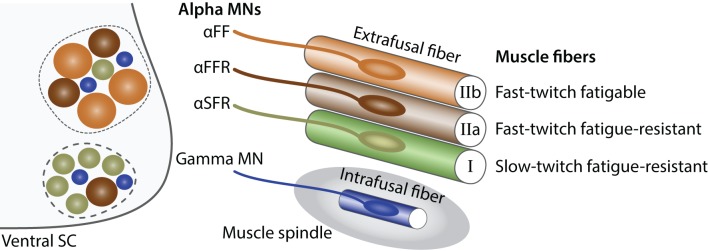
**Characteristics of alpha and gamma MNs**. Schematic showing the principal characteristics of alpha and gamma MNs (adapted from Kanning et al., 2010). Within the ventral spinal cord (SC light gray), MN pools (dashed lines) are composed of gamma MNs (blue) as well as three type of alpha MNs: αFF (light brown), αFFR (dark brown), αSFR (green). Alpha MNs have a larger diameter than gamma MNs. Beta MNs are not represented for simplicity. The proportion of alpha MN subtypes varies between MN pools. In the periphery, a muscle is composed of three types of extrafusal fibers: fast-twitch fatigable muscle fibers (light brown, IIb) are innervated by αFF MNs, fast-twitch fatigue-resistant muscle fibers (dark brown, IIa) are innervated by alpha αFFR MNs and slow-twitch fatigue-resistant muscle fibers (green, I) are innervated by αSFR MNs. Intrafusal muscle fibers (blue) reside within a muscle spindle (gray) and are exclusively innervated by gamma MNs. A single MN innervate multiple fibers all of the same type; however, for the schematic simplicity only one fiber is represented.

***Alpha motor neurons***. Alpha MNs exclusively innervate extrafusal muscle fibers and are the key of muscle contraction (Figure 1). Anatomically, alpha MNs are characterized by a large cell body and a well-characterized neuromuscular ending. They have an important role in the spinal reflex circuitry by receiving monosynaptic innervation directly from SNs thus minimizing the delay of the response (Eccles et al., 1960). Alpha MNs can be further divided into 3 different subtypes depending on the extrafusal fiber type they innervate: (i) SFR, (ii) FFR, and (iii) FF (Burke et al., 1973) (Figure 2). There is no universal criteria distinguishing alpha MNs subtypes; however, some trends are observed in term of size, excitability, and firing pattern. SFR MNs tend to have a smaller cell body diameter and thus a higher input resistance making them responsive to a lower stimulation threshold. As a result, SFR MNs are recruited first during muscle contraction. They also have the capacity of maintaining a persistent activity even after the stimulation ceased (Lee and Heckman, 1998). On the other hand, FF MNs have often a larger cell body and are firing after the initial recruitment of SFR neurons giving extra strength to the activated muscle. In terms of conduction velocity, MNs innervating fast fibers are substantially faster (100 m/s) than SFR MNs (85 m/s) (Burke et al., 1973). Lastly, little is known about FFR MNs physiology; yet, they are considered to have intermediate characteristics between FF and SFR MNs (Figure 2).

**Figure 3:**
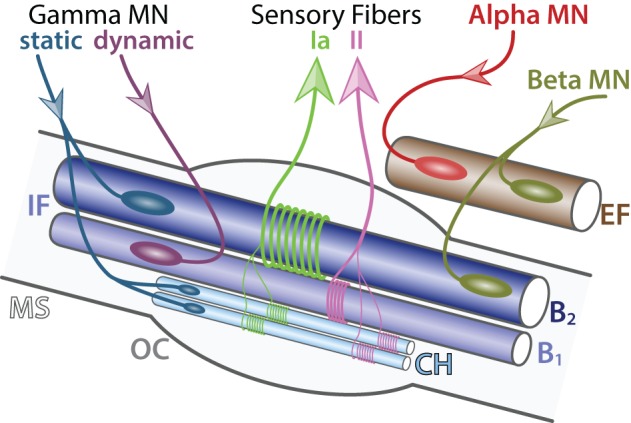
**Detailed innervation of a muscle spindle**. Schematic of an adult muscle spindle (MS, light gray) on the longitudinal section (adapted from Maier, 1997). Alpha MN (red) exclusively innervates (incoming arrow) extrafusal fibers (EF, brown). Beta MNs (green-brown) innervate both EF and intrafusal fibers (IF, blue). Gamma MNs are divided into two subtypes: static (blue) connecting to nuclear chain (CH, light blue) and nuclear bag 2 (B2, dark blue) fibers and dynamic (purple) connecting to nuclear bag 1 fibers (B1, intermediate blue). Sensory afferent axons Ia (light green) and II (pink) convey information (outgoing arrows) to sensory neurons located in the dorsal root ganglia. The outer capsule (OC) is a dedicated membrane isolating the muscle spindle from the extrafusal fibers. A single MN innervate multiple fibers all of the same type; however, for the schematic simplicity only one fiber is represented.

***Beta motor neurons***. Beta MNs are smaller and less abundant than other somatic MN subtypes. As a result beta MNs are poorly characterized. They innervate both intrafusal and extrafusal muscle fibers (Bessou et al., 1965) (Figure 3). Therefore, beta MNs constitute an exception to the homogeneity observed in motor-units and control both muscle contraction and responsiveness of the sensory feedback from muscle spindles. They are further subdivided into two subtypes depending on the type of intrafusal fibers they innervate: (i) static, innervating nuclear chain fibers and (ii) dynamic, innervating the nuclear bag fibers of muscle spindles. Static beta MNs increase the firing rate of type Ia and type II sensory fibers at a given muscle length whereas dynamic beta MNs increase the stretch-sensitivity of the type Ia sensory fibers by stiffening the nuclear bag fibers. Beta MNs are mainly characterized anatomically and functionally, further molecular and electrical properties remain to be identified.

***Gamma motor neurons***. Gamma MNs control exclusively the sensitivity of muscle spindles. Their firing increases the tension of intrafusal muscle fibers and therefore mimics the stretch of the muscle. Like beta MNs, gamma MNs are functionally divided into two subtypes: (i) static, innervating nuclear chain fibers and static nuclear bag fibers and (ii) dynamic, innervating the dynamic nuclear bag fibers (Figure 3). Gamma MNs receive only indirect sensory inputs and do not possess any motor function. Therefore, gamma MNs do not directly participate to spinal reflexes (Eccles et al., 1960) but instead contribute to the modulation of muscle contraction.

### Summary of motor neuron classification

As seen above, the term “motor neuron” groups a significant diversity of cell types and does not ideally reflect biological reality. Upper and lower MNs are fundamentally different and their shared nomenclature can easily be misleading. For instance, if we define a MN by being a “neuronal cells settling within but projecting outside of the CNS,” upper MNs would be excluded. In fact, upper MNs would be more accurately defined by the terminology “imbuo-neurons” derived from Latin *imbuo* that signifies “give initial instruction” or by the terminology “didactic-neurons” derived from the Greek *didaktikós* for instructive. In contrast, lower MNs, with the exception of visceral MNs, connect directly to their muscle targets and constitute the last step of the neuronal circuitry. SpMNs are divided into functional groups, termed pools, mirroring the diversity of muscle targets in the periphery. In addition, a single muscle is composed of several fiber types that are innervated by specific classes of MNs. Therefore MN pools should not be considered as a set of identical cells but instead as a mosaic of MN cell types covering a broad range of functions. The generation of this complex architecture must rely on precise mechanisms ensuring the establishment of the correct connections between matching MN - target pairs. We will review the functional organization of SpMNs as well as the molecular mechanisms leading to their generation.

## Generation of spinal motor neurons

**Figure 4:**
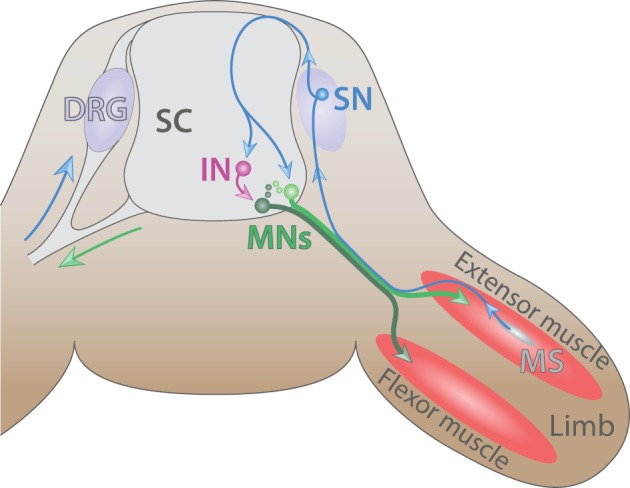
**The spinal cord reflex circuitry**. Schematic of a myotatic reflex illustrating the spinal cord (SC) circuitry (adapted from Purves and Williams, 2004). Sensory neuron (SN, blue) located in the dorsal root ganglia (DRG) transmits a stretch stimulus sensed by the muscle spindle (MS, gray) to an interneuron (IN, purple) as well as directly to motor neurons (MNs, dark and light green). In turn, MNs stimulate the contraction of extensor muscle (red) and ensure the concomitant relaxation of the antagonist flexor muscle located in the limb.

The spinal cord offers a relatively simple, yet, powerful experimental model to study neuronal development. It can be schematized as a circuitry formed by three different neuron types. Sensory neurons located in the dorsal root ganglia (DRG) receive input information from the periphery and transmit it either directly to alpha MNs located in the ventral horn (monosynaptic connections) or to association neurons (commissural and interneurons) that, in turn, process and convey the information toward the MNs. MNs then stimulate their respective effector that will generate the appropriate output response (Eccles et al., 1957) (Figure 4). Over the last three decades, many studies have shaded light on important mechanisms governing MN differentiation in the spinal cord. A comprehensive and up-to-date review of those studies will be presented below.

### Developmental origin

**Figure 5:**
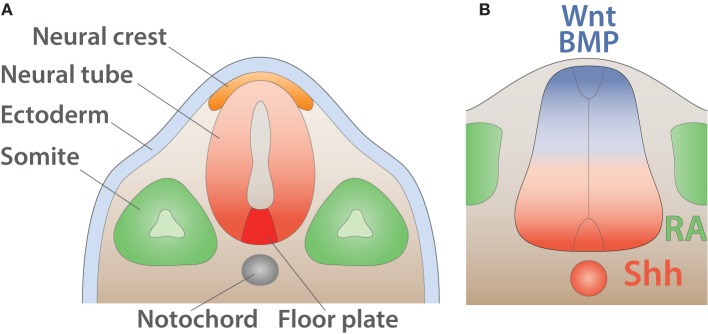
**Early anatomy and inductive signals in the neural tube**. **(A)** Schematic of the anatomy of the neural tube after neurulation (adapted from Purves and Williams, 2004). The ectoderm (light blue) is positioned on the external side whereas neural crest (orange) resides underneath. The notochord (gray) induces the differentiation of the floor plate (red). The somites (green) give rise to muscles and bones. **(B)** Schematic summarizing signals involved in the dorso-ventral pattering of the mouse neural tube shown in transverse section (adapted from Dessaud et al., 2008). Wnt and BMP secreted by the roof plate (blue) as well as retinoic acid (RA) produced by the somites (green) cooperate with Shh expressed by the floor plate and the notochord (red) to pattern the neural tube.

During the early phase of embryogenesis, the egg cell undergoes a series of divisions until forming a sphere made of a single layer of cells called the blastula. Subsequently, during a process called gastrulation, a group of cells will enter the blastula cavity leading in triploblastic animals to the formation of the three primary germ layers: (i) the endoderm, (ii) the mesoderm, and (iii) the ectoderm. Individual layers generate progenies restricted to a limited number of distinct fates. The ectoderm undergoes a process called neurulation in which it folds inward and leads to the formation of three ectodermic masses: (i) the neural tube, (ii) the neural crest cells, and (iii) the external ectoderm. The external ectoderm generates the epidermis whereas the neural crest cells form the peripheral ganglion, the pigments of the skin as well as the dorsal root ganglia. Finally, the neural tube gives rise to the CNS, composed of the brain and the spinal cord (Purves and Williams, 2004) (Figure 5A).

### Generation of dedicated spinal cord progenitor domains

Soon after neurulation, the neural tube is surrounded by several inductive signals stimulating the subsequent differentiation process. Members of the wingless-type MMTV integration site family (WNT) (Alvarez-Medina et al., 2008) and of the bone morphogenetic protein family (BMPs) (Mehler et al., 1997) and their regulators Noggin (NOG), Chordin (CHRD), and Follistatin (FST) (Zimmerman et al., 1996; Streit et al., 1998) are expressed in a decreasing dorsal to ventral gradient. Additionally, the surrounding paraxial mesoderm expresses the aldehyde dehydrogenase 1 A2 (ALDH1A2 or RALDH2) (Niederreither et al., 1997), which converts retinaldehyde into retinoic acid (RA) a well-characterized regulator of neuronal differentiation (Pierani et al., 1999; Novitch et al., 2003; Lee et al., 2009). Together those signals collaborate with an increasing ventral to dorsal gradient of sonic hedgehog morphogen (SHH) secreted by the underlying notochord as well as the floor plate (Yamada et al., 1991, 1993; Roelink et al., 1994; Marti et al., 1995a,b; Ericson et al., 1996) (Figure 5B).

**Figure 6:**
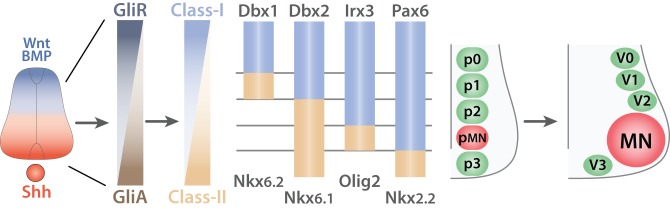
**Generation of ventral spinal progenitor domains**. Schematic summarizing the mechanisms of progenitor domain formation in the ventral spinal cord (adapted from Ulloa and Marti, 2010). Opposing gradients of Shh (red) and Wnt/BMP proteins (blue) are transduced into Gli protein activity. Gli activators (GliA, brown) in the most ventral region induce the expression of Class-II proteins (light brown) whereas Gli repressors (GliR, dark gray-blue) induce Class-I proteins (light blue) in the dorsal portion of the ventral spinal cord. This initial expression pattern is subsequently refined by cross-repressive interactions between pairs of Class-I and Class-II proteins to generate five exclusive progenitor domains (p0, p1, p2, p3, and pMN). V0, V1, V2, V3, interneurons arise from the p0, p1, p2, and p3 respectively whereas all MNs derive from the pMN progenitors.

Molecularly, SHH binds to the patched homolog 1 receptor (PTCH1) (Stone et al., 1996) and releases its constitutive inhibition of the smoothened homolog (SMO) (Quirk et al., 1997) thereby, preventing the degradation of the GLI-Kruppel family (GLI) proteins (Chen et al., 2011b; Niewiadomski et al., 2014). Hence, SHH signaling correlates directly with GLI activity (GliA) (Figure 6). Conversely, signals from the roof plate induce the expression of GLI repressors (GliR). Together, ventral and dorsal signals lead to a net decreasing gradient of GLI activity from the ventral to the dorsal. In turn, GLI proteins promote or repress in a concentration dependent manner homeodomain transcription factors that can be sorted into two classes: (i) Class-I; paired box 3/6/7 (PAX3/6/7), developing brain homeobox 1 and 2 (DBX1/2), and Iroquois related homeobox 3 (IRX3) are repressed by GliA and thus expressed dorsally whereas (ii) Class-II NK2 homeobox 2 and 9 (NKX2.2/2.9), NK6 homeobox 1 and 2 (NKX6.1/6.2), and oligodendrocyte transcription factor 2 (OLIG2) are induced by GliA and therefore, located ventrally (Shirasaki and Pfaff, 2002). This initial patterning is subsequently refined by cross-repression between pairs of class-I and class-II proteins. Studies using systematic gain or loss of function approaches have identified the specific pairs of class-I and class-II proteins. For example, inactivation of OLIG2 leads to a ventral expansion of IRX3 (Zhou and Anderson, 2002) whereas ectopic expression of NKX6.1 restrains the expression of DBX2 to the most dorsal domain (Briscoe et al., 2000). Thus, cross-repressive interactions between pairs of class-I and class-II proteins guarantee the formation of sharp boundaries between adjacent domains and ensure that they remain mutually exclusive. Ultimately, this process leads to the emergence of five ventral progenitor domains (p0, p1, p2, pMN, and p3) defined by the expression of a unique combination of transcription factors (Ericson et al., 1997; Briscoe et al., 2000; Vallstedt et al., 2001). This simple mechanism is in fact more complex as additional molecules ensure the integrity of each individual progenitor domains. For example both WNT signaling pathway (Lei et al., 2006; Alvarez-Medina et al., 2008; Yu et al., 2008) and the transducin-like enhancer of split 1 (TLE1) (Todd et al., 2012) contribute to reinforce the ventral boundary of the pMN domain. Another level of complexity arises from the interpretation of SHH gradient that is modulated by the downstream network of transcription factors. Hence, tis mechanism creates a feedback loop during the developmental period allowing the modulation of progenitor domain formation (Balaskas et al., 2012).

Ultimately, the five ventral progenitor domains will generate neuronal cells restricted to a specific lineage (V0, V1, V2, V3, INs, and MNs) (Alaynick et al., 2011). Conceptually, the strategy used for the establishment of the progenitor domains involves inductive gradients interpreted into the expression of specific combinations of transcription factors. Cross-repressive interactions between pairs of transcription factors ensure the creation of mutually exclusive domains. Each progenitor domain then generates progenies restricted to a specific lineage.

### Acquisition of motor neuron fate

All SpMNs arise from the unique pMN progenitor domain that expresses the unique combination of the homeodomain proteins NKX6.1, PAX6, and the basic helix-loop-helix (bHLH) protein OLIG2 (Tanabe et al., 1998; Novitch et al., 2001; Vallstedt et al., 2001). To become a mature MN, progenitors need to exit the cell cycle and enter the differentiation process. These events must be tightly regulated in order to generate an appropriate number of differentiated cells at a particular time during neurogenesis. Several mechanisms involved these transitions have been characterized and will be described here.

First, RA described previously as a regulator of progenitor domain formation, is also involved in the acquisition of the MN fate (Novitch et al., 2003). This process illustrates a principle commonly seen in developmental biology and in biology in general, namely, the use of a single cue at multiple steps during development as a mean to reduce the biological cost in energy. RA induces in MN progenitors the expression of glycerophosphodiester phosphodiesterase domain containing 5 (GDPD5 or GDE2) (Jacobson and Rao, 2005; Rao and Sockanathan, 2005). In turn, GDPD5 complexes with the peroxiredoxin 1 (PRDX1) (Yan et al., 2009) and with the GDP form of the G protein alpha subunit i2 (GNAI2) (Hammerle and Tejedor, 2007; Periz et al., 2010; Sabharwal et al., 2011; Park et al., 2013) to promote the MN differentiation program. Similarly, the cut-like homeobox 2 (CUX2) is involved in progenitors' cell cycle progression and cell cycle exit (Iulianella et al., 2008).

In parallel, OLIG1 and 2 contribute to the expression of another bHLH protein named neurogenin 2 (NEUROG2) (Sommer et al., 1996; Novitch et al., 2001, 2003; Scardigli et al., 2001; Lu et al., 2002; Zhou and Anderson, 2002; Lee and Pfaff, 2003; Lee et al., 2004). NEUROG2 interacts with the RA receptor (RAR) and recruits the histone acetyl transferases CREB binding protein (CREBBP) and E1A binding protein p300 (EP300) (Lee et al., 2009) to promote the transcription of downstream MN genes (Mizuguchi et al., 2001; Novitch et al., 2001; Scardigli et al., 2001; Lu et al., 2002; Zhou and Anderson, 2002; Lee and Pfaff, 2003). Interestingly, during the early stage of MN generation OLIG2 and NEUROG2 collaborate to promote MN fate (Mizuguchi et al., 2001). At later stages, the persistence of OLIG1/2 expression and the concomitant down-regulation of NEUROG2 allow the emergence of oligodendrocyte progenitors from the pMN domain (Richardson et al., 2000; Lu et al., 2002; Zhou and Anderson, 2002). Therefore, the dynamic regulation of OLIG2 and NEUROG2 during neurodevelopment allows the sequential generation of MNs and oligodendrocytes at different time from a common progenitor domain (Lee et al., 2005). An important downstream target of OLIG2 and NEUROG2 signaling is the motor neuron and pancreas homeobox 1 (MNX1 or HB9) (Tanabe et al., 1998; Lee et al., 2005, 2009). Remarkably, MNX1 stimulates its own expression (Tanabe et al., 1998) providing to developing MNs their independence from SHH and RA signaling. Therefore, MNX1 has been used as a reliable and specific marker of post-mitotic SpMNs.

Although cell fates seem to be established early in development, some evidences suggest that additional mechanisms that ensure their maintenance are required. For example, MNs and V2 INs are generated by two adjacent progenitor domains (Figure 6). Inactivation of MNX1 in developing MNs induces a switch toward V2 IN fate (Arber et al., 1999; Thaler et al., 1999). Comparably, the runt-related transcription factor 1 (RUNX1), whose expression is restricted to selected post-mitotic cervical MNs (Theriault et al., 2004; Stifani et al., 2008; Guizard et al., 2010) is important for the consolidation of MN phenotype by ensuring the persistent suppression of the IN program (Stifani et al., 2008). The molecular mechanism underlying the divergence between V2 INs and MNs have been remarkably revealed by Pfaff and colleagues and involves the transient expression of the LIM homeobox 3 (LHX3) in developing MNs and V2 INs (Thaler et al., 2002). In prospective V2 INs, LHX3 forms a complex with the LIM domain binding 1 (LDB1 or NLI) and promotes the IN fate via the LIM domain only 4 (LMO4) (Thaler et al., 2002; Lee et al., 2008). In prospective MNs, the ISL1 transcription LIM homeodomain (ISL1) is induced by SHH secreted by the notochord and floor plate (Yamada et al., 1991; Ericson et al., 1992) and inserts into the LHX3-LDB1 complex to induce a switch toward MN specification (Thaler et al., 2002; Song et al., 2009). Most importantly ISL1-LHX3 complex directly binds and induce the expression genes involved in cholinergic neurotransmission, a fundamental characteristic of SpMNs (Cho et al., 2014; Kania, 2014a). Despite these important findings the acquisition and the maintenance of MN fate remain to be fully understood. A recent study has shed light on the mechanisms by which MN precursors detach from neuroepithelium and migrate laterally as they exit the cell cycle (Rousso et al., 2012). What are the molecular mechanisms controlling such migration? Rousso et al. (2012) remarkably identify the role of the forkhead box P2 and 4 (FOXP2/4) in promoting the detachment of newly born MNs from the ventricular zone. Additionally, the authors elegantly linked nuclear gene regulation to effector protein at the membrane. Namely, FOXP2/4 repress the expression cadherin 2 (CDH2) responsible for the attachment of developing progenitors to the neuroepithelium.

In conclusion, the emergence of newborn MNs from the pMN progenitor domain relies on the precise control of the balance between proliferation and differentiation. Although OLIG2 and NEUROG2 have prominent roles into MN fate commitment, additional mechanisms are required to ensure the consolidation of this phenotype. Following the acquisition of their general identity, MNs need to differentiate and acquire features required for their respective function. This process, termed patterning, will be described hereafter.

### Patterning in spinal motor neuron development

Following the initial acquisition of their general fate, newborn SpMNs are required to further differentiate to adopt an identity reminiscent of their respective muscle targets. The general strategy is, at least in part, comparable to the mechanisms leading to the emergence of spinal progenitor domains. Globally, SpMN specification follows a temporal gradient along the ventro-dorsal and rostro-caudal axes (Nornes and Carry, 1978). MNs located more ventrally and more rostrally are generated earlier. This temporal regulation reflects two mechanisms: (i) the progressive expansion of the total volume of the neural tissue and (ii) the generation of specific cell types along the rostro-caudal axis.

Several proteins including the fibroblast growth factor (FGF), the growth differentiation factor 11 (GDF11 or BMP-11), members of transforming growth factor beta (TGFB) family as well as RA (Durston et al., 1989; Muhr et al., 1999; Liu et al., 2001; Bel-Vialar et al., 2002; Sockanathan et al., 2003; Liu, 2006) form a gradient along the rostro-caudal axis and induce, in a concentration-dependent manner, the expression of protein of the homeobox (HOX) family (Ensini et al., 1998; Lance-Jones et al., 2001). *Hox* genes are arranged into genomic clusters and their response to FGF and RA concentration is correlated to their position within a cluster (Liu et al., 2001; Bel-Vialar et al., 2002). Genes located at the 5′ end are induced by high concentration of FGF and thus expressed in more caudal regions. Conversely, genes at the 3′ end are induced by low concentrations of FGF and therefore expressed in more rostral regions (Liu et al., 2001; Bel-Vialar et al., 2002; Dasen et al., 2003; Mazzoni et al., 2013b). After the initial activation of HOX protein expression, further refinement is achieved at the rostral boundary by histone modifications performed by the Bmi1 polycomb ring finger oncogene (BMI1) part of the polycomb repressive complex (Golden and Dasen, 2012). At the caudal edge, cross-repressive interactions between pairs of HOX proteins (Dasen et al., 2003, 2005) lead to non-overlapping domains. For instance, HOX6, 9, and 10 expression correlates with the brachial, thoracic, and lumbar segments, respectively.

Subsequently, HOX patterning induces the formation of anatomical columns termed motor columns along the rostro-caudal axis (Shah et al., 2004; Dasen et al., 2005; Jung et al., 2010). The underlying mechanisms have been partially defined since HOX patterning converges toward the expression of FOXP1 (Arber, 2008; Dasen et al., 2008; Pfaff, 2008; Rousso et al., 2008; Palmesino et al., 2010). Mechanistically, HOX6 and 10 at brachial and lumbar segments respectively direct the expression of FOXP1, which in turn cooperate with HOX proteins to induce the formation of limb specific MNs at the expense of thoracic MNs (Dasen et al., 2008; Rousso et al., 2008). Additionally, HOXC9 has a critical role in restricting appendage specific MNs to the limb innervating segments by selectively excluding them from thoracic segments (Jung et al., 2010, 2014). This effect is at least partially mediated by direct and indirect repression of FOXP1 in thoracic segments.

In summary, after the formation of dedicated progenitor domains, intrinsic and extrinsic molecules cooperate to promote a general MN fate. Inductive signals along the rostro-caudal axis profile developing MNs to adjust to specific local needs. Together these mechanisms lead to the formation of anatomically defined motor columns. We will describe hereafter each column by providing information on their molecular specificity as well as mechanisms of their formation.

### Columnar organization of spinal motor neurons

**Figure 7:**
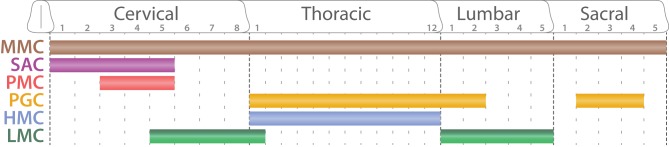
**Segmental organization of spinal motor columns**. Schematic summarizing the segmental distribution of spinal motor columns (adapted from Dasen and Jessell, 2009). While the medial motor column (MMC, brown) is present all along the rostro-caudal axis, the spinal accessory column (SAC, purple) is restricted to the five first cervical segments (C1–C5). The phrenic motor column (PMC, red) is confined between C3 and C5. The preganglionic column (PGC, orange) extends through the thoracic segments until the second lumbar segments (L2) as well as well as between sacral segments 2 and 4 (S2–S4). The hypaxial motor column (HMC, light blue) is exclusive of the thoracic segment where as the lateral motor column (LMC, dark and light green) is located at limb levels: brachial (C5-T1) and lumbar segments (L1–L5).

**Figure 8:**
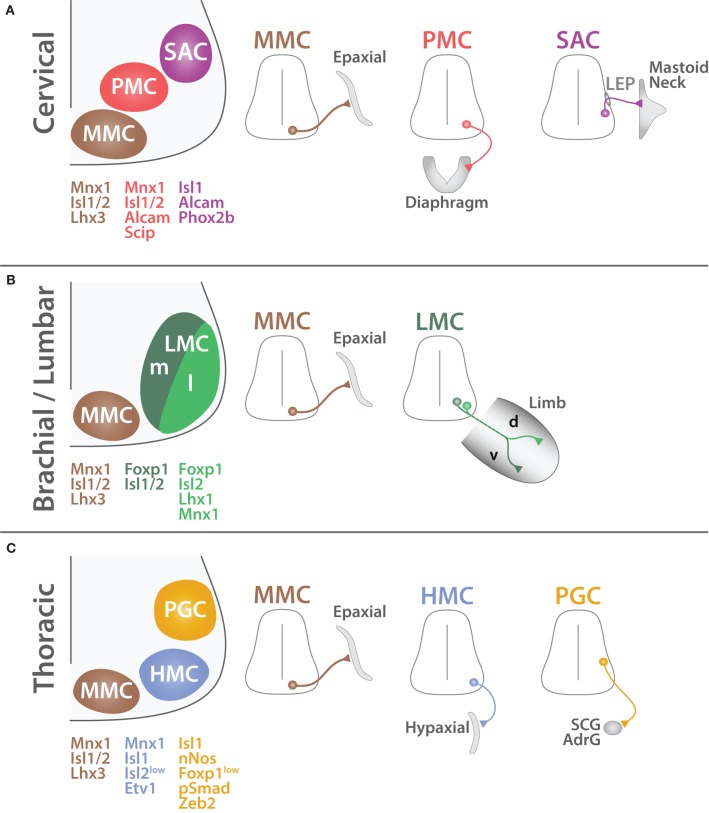
**Organization of SpMNs at cervical, brachial/lumbar and thoracic levels**. Schematic summarizing the characteristics of spinal motor columns at cervical **(A)**, brachial/lumbar **(B)** and thoracic **(C)** levels (adapted from Dasen and Jessell, 2009). MMC MNs (brown) are located medially and connect to the axial musculature (Epaxial). PMC MNs (red) have an inter-medio-lateral position and connect to the diaphragm. SAC MNs (purple) exit the CNS via the lateral exit point (LEP) and connect to mastoid and neck muscles. LMC MNs (green) are divided into two divisions medial (m, dark green) and lateral (l, light green). LMCm MNs connect to the ventral (v) part of the limb whereas LMCl MNs innervate the dorsal (d) region. HMC MNs (light blue) are located in the medio-lateral region and connect to the body wall and intercostal muscles (Hypaxial). PGC MNs (orange) are positioned dorso-laterally and innervate to the sympathetic chain ganglia (SCG) and chromaffin cells of the adrenal gland (AdrG). Proteins expressed by each column are depicted with their respective color code.

SpMNs are organized into distinct anatomical columns extending along the rostro-caudal axis and called motor columns (Figure 7). Previous studies have described four main columns: the median motor column (MMC), the lateral motor column (LMC), the hypaxial motor column (HMC), and the preganglionic column (PGC) (Prasad and Hollyday, 1991; Tsuchida et al., 1994; Jessell, 2000; Alaynick et al., 2011; Francius and Clotman, 2014) Each column possesses a coherent gene expression profile as well as a uniform axonal projection pattern (Figure 8). We will hereafter describe their molecular identity as well as the developmental mechanisms required for their formation. Moreover, we will complete this picture by describing the less well-characterized spinal accessory column (SAC) and the phrenic motor column (PMC).

#### The median motor column

MMC MNs are located in the medial region of the ventral spinal cord and target to the dermomyotome (Gutman et al., 1993; Tsuchida et al., 1994), which gives rise to the axial musculature later in development (Fetcho, 1987; Gutman et al., 1993). Axial muscles are mainly involved in the maintenance of the body posture and are found all along the body axis. Therefore, MMC MNs are not segmentally restricted and are found all along the spinal cord (Figure 7). MMC MNs are characterized by the co-expression of MNX1, ISL1/2, and LHX3 (Tsuchida et al., 1994) (Figure 8). In mature MNs, LHX3 is unique to MMC MNs and its forced expression is sufficient to impose MMC identity (Sharma et al., 1998, 2000). LHX3 is therefore commonly used as a reliable marker of MMC MNs; however, as mentioned earlier, LHX3 is also transiently expressed developing MN in which it contributes to the establishment of their identity at the expense of the V2 INs. Interestingly, MMC MNs present an exception to the rostro-caudal patterning of HOX proteins. How do MMC MNs escape HOX rostro-caudal patterning? Molecularly, proteins from the WNT family (WNT4/5A/5B) are expressed in a ventral to dorsal decreasing gradient (Agalliu et al., 2009) and permit the persistence of LHX3/4 expression (Shirasaki and Pfaff, 2002; Agalliu et al., 2009) in the most ventral region. In turn, LHX3/4 make MMC MNs unresponsive to HOX patterning (Dasen et al., 2005, 2008). As suggested by Dasen and colleagues, this unique feature likely reflects the ancestral properties of the MMC from which other motor column have derived during evolution (Dasen et al., 2008; Dasen, 2009; Dasen and Jessell, 2009; Jung et al., 2010; Philippidou and Dasen, 2013).

#### The spinal accessory column

SAC MNs are located in the intermedio-lateral region of the spinal cord and expand from the end of the medulla until the 5th cervical segment (C1–C5) (Jacobson and Marcus, 2007; Ullah et al., 2007) (Figure 7). SAC MNs innervate mastoid muscles as well as four muscles of the neck (*Sternomastoid, Cleidomastoid, Cleidotrapezius, and Acromiotrapezius*) (Brichta et al., 1987; Watanabe and Ohmori, 1988). While SAC MNs innervating the mastoid muscles are located in the rostral portion, MNs innervating the *trapezius* muscles are located in the most caudal segments of the C1–C5 segment of the spinal cord (Ullah et al., 2007; Stifani et al., 2008). SAC MNs are different from other SpMNs because they innervate muscles that derive from branchial arches (Pabst et al., 1998; Aldskogius et al., 2009) and because their axons penetrate the periphery by exiting through the lateral exit point (LEP) located midway along the dorso-ventral axis of the spinal cord (Sharma et al., 1998; Schubert and Kaprielian, 2001; Pabst et al., 2003; Dillon et al., 2005; Bravo-Ambrosio and Kaprielian, 2011) (Figure 8). Therefore, SAC MNs are also referred as dorsal MNs (dMNs) as opposed to ventral MNs (vMNs) exiting classically via the ventral root. Molecularly, SAC MNs have been successfully distinguished from other MNs by the use of different markers such as activated leukocyte cell adhesion molecule (ALCAM or BEN) (Schubert and Kaprielian, 2001; Dillon et al., 2005) as well as ISL1, RUNX1 and the paired-like homeobox 2b (PHOX2B) (Pattyn et al., 2000; Amiel et al., 2009; Dubreuil et al., 2009; Stifani and Ma, 2009; Kobayashi et al., 2013; Mazzoni et al., 2013a; Laumonnerie et al., 2014).

Developmentally, the NK2 homeobox 9 (NKX2.9) has been shown to be required for proper SAC MN generation (Pabst et al., 2003) as well as for SAC axonal projection (Dillon et al., 2005). Conversely, LHX3/4 inactivation leads to an increase number of SAC MNs (Sharma et al., 1998) whereas LHX3 is sufficient to promote vMNs at the expense of dMNs (Lieberam et al., 2005; Hirsch et al., 2007; Kobayashi et al., 2013). Together these results suggest that the expression of LHX3/4 allows vMNs generation at the expense SAC MNs.

SAC MNs are a very peculiar population of SpMNs as they are the exclusive cells of branchial type in the spinal cord. Several characteristics described above are reminiscent of hindbrain branchiomotor and visceromotor MN populations and largely differ from other SpMNs. SAC MNs may appear therefore as a transitional population between the hindbrain and cervical MNs.

#### The phrenic motor column

Phrenic MNs are located in the cervical segments C2–C6 at embryonical stages and become progressively confined between cervical levels C3–C5 by birth (Webber and Pleschka, 1976; Allan and Greer, 1997a,b; Song et al., 2000) (Figure 7). They connect to a particular muscle: the diaphragm. This muscle is essential for respiration and therefore is under constant rhythmic activity. This characteristic also applicable to the cardiac muscle differs diametrically from skeletal muscles required to generate unsystematic contraction. The diaphragm is involved in both inspiration and expiration, both conscious and unconscious. Because of its unique function, phrenic MNs are required to produce a perpetual rhythmic firing as early as the first instant after birth and throughout life. Although phrenic MNs have been well characterized in terms of cell body position and anatomical properties, until recently little was known about their molecular characteristics. In a well-detailed study, Philippidou and colleagues identified for the first time the molecular profile of phrenic MNs (Philippidou et al., 2012). Namely, phrenic MNs are under the control of HOX5 patterning and are expressing high levels of the POU domain class 3 transcription factor 1 (POU3F1 or SCIP) as well as ISL1/2, and MNX1 (Thaler et al., 1999; Rousso et al., 2008; Castellani and Kania, 2012; Philippidou et al., 2012; Philippidou and Dasen, 2013) (Figure 8). Interestingly, soon after birth, phrenic MNs as well as the diaphragm muscle itself undergo significant anatomical and functional transformations including dendritic arborization and electrical activity properties (Cameron et al., 1991; Prakash et al., 2000). These changes reflect the passage from the aquatic intrauterine gestation to the aerobic life. Therefore, although phrenic MNs are established early in development to ensure their functionality for the time of birth, additional mechanisms, yet to be characterized, are likely occurring after birth to guarantee further post-natal maturation. The vital role of the PMC coupled to the recent molecular findings cited above encouraged Machado and colleagues to induce the differentiation of embryonic stem cells into phrenic MNs *in vitro* (Machado et al., 2014). This prodigious achievement carries great hope for stem cell based regenerative therapies as patients suffering from SpMN diseases ultimately face respiratory impairments. Nevertheless, additional efforts are still required to establish a viable therapy from this initial breakthrough.

#### The preganglionic motor column

PGC MNs also known as spinal visceral MNs constitute the CNS component of the ANS. They are located in the thoracic and upper lumbar spinal segments (T1–L2) (Figure 7) where they occupy an intermedio-lateral location (Figure 8). They do not innervate skeletal muscles as other somatic SpMNs do but instead connect to the sympathetic ganglia. Thus, PGC MNs are involved in stimulation of smooth muscles as well as in control of glands secretions. They can be molecularly identified by the expression of the SMAD family member 1 (SMAD1 or pSMAD1) (Dasen et al., 2008), nitric oxide synthase 1 neuronal (NOS1 or nNOS) (Saito et al., 1994; Wetts and Vaughn, 1994; Dasen et al., 2003), zinc finger E-box binding homeobox 2 (ZEB2 or SIP1) (Roy et al., 2012), as well as low level of FOXP1 (Dasen et al., 2008; Rousso et al., 2008; Morikawa et al., 2009).

Despite extreme functional differences, somatic and visceral SpMNs arise from common precursors expressing ISL1/2 (Prasad and Hollyday, 1991; Thaler et al., 2004). The maintenance of ISL2 in maturing MNs leads to the generation of somatic MNs whereas its down-regulation together with the persistence of ISL1 guides maturing MNs toward the visceral fate (Thaler et al., 2004). Recently, the one cut domain family members (ONECUT1/2/3), expressed in newly born MNs (Francius and Clotman, 2010, 2014; Audouard et al., 2012), have been found to bind directly to a specific enhancer region of *Isl1* gene to maintain its expression (Roy et al., 2012) resulting in a limitation of PGC MN formation. This consequence is challenged by the effect of ZEB2 promoting PGC formation. Therefore, opposing and cooperating mechanisms ensure the proper divergence between somatic and visceral SpMNs.

As mentioned earlier, spinal visceral MNs are also found in the sacral segments (S2–S4). However, these cells belong to the parasympathetic system (rest and digest) while thoracic PGC MNs belong to the sympathetic autonomic system (fight or flight). In addition to these functional differences, thoracic and sacral PGC MNs also differ in terms of axonal projections. While thoracic PGC MNs connect to the sympathetic chain ganglia located in the proximity of the spine, sacral PGC MNs connect to ganglia in the vicinity of the effector targets (kidney, bladder, gonads). Therefore, the molecular properties of sacral PGC MNs that remain largely unknown are presumably substantially different from thoracic PGC MNs.

#### The hypaxial motor column

Initially the MMC had been separated in two divisions: (i) a medial MMC (MMCm), described above as MMC, targeting to axial musculature and present all along the rostro-caudal axis and (ii) a lateral MMC (MMCl) targeting to the body wall and present only in the thoracic segments (Gutman et al., 1993; Jessell, 2000). However, recent molecular findings have associated MMCl MNs with PGC and LMC MNs rather than with MMC MNs (Dasen et al., 2008; Rousso et al., 2008). Therefore, the MMCl has been referred to as the hypaxial motor column (HMC) (Dasen et al., 2008; Agalliu et al., 2009). This new nomenclature better reflects HMC MN molecular nature and avoids confusion with MMC MNs. HMC MNs are located in the ventro-lateral spinal cord and innervate muscles derived from the ventral mesenchyme (Smith and Hollyday, 1983). The ventral mesenchyme gives rise to the body wall musculature composed of the intercostal and abdominal muscles present only at thoracic level (Prasad and Hollyday, 1991). Therefore, HMC MNs are only present at thoracic level (Tsuchida et al., 1994; Sharma et al., 2000) (Figure 7). Molecularly, HMC MNs are characterized by the expression of MNX1, ISL1, ETS variant 1 (ETV1 or ER81) and low levels of ISL2 (Dasen et al., 2008; Rousso et al., 2008) (Figure 8). Interestingly, FOXP1 inactivation converts both PGC and LMC MNs to a HMC phenotype (Dasen et al., 2008). As suggested by Dasen and Jessell (2009), HMC and MMC MNs likely reflect the vestige of an ancestral spinal motor column organization from which other motor columns derived (Jung et al., 2014). Finally, because intercostal and abdominal muscles are involved in respiration, HMC MNs could presumably be somehow related to PMC MNs described previously. To our knowledge no experiment has been reported to address this suggestion that remains to be tested.

#### The lateral motor column

LMC MNs are located in the most lateral portion of the ventral spinal cord (Bueker, 1944). They connect to muscles of the appendages and therefore are present only at limb levels also defined as brachial (C5 to T1) and lumbar levels (L1–L5) (Hollyday and Hamburger, 1977; Hollyday and Jacobson, 1990) (Figure 7). This segmentation reflects the rostro-caudal patterning of HOX proteins (Kessel and Gruss, 1991; Liu et al., 2001; Dasen et al., 2003) controlled by local inductive signals (Ensini et al., 1998).

LMC MNs are further separated into two divisions: medial and lateral (Tosney et al., 1995). These divisions retain a topographic correspondence with the localization of their target in the periphery. Medial LMC (LMCm) MNs target to the ventral part of the limb whereas lateral LMC (LMCl) MNs innervate the dorsal limb musculature (Landmesser, 1978; Tosney and Landmesser, 1985a,b; Kania et al., 2000) (Figure 8). Molecularly, LMC MNs are characterized by the expression of ISL2, FOXP1, and ALDH1A2 and do not sustain LHX3 expression (Tsuchida et al., 1994; Sockanathan and Jessell, 1998; Dasen et al., 2008; Rousso et al., 2008). Sockanathan and Jessell (1998) have remarkably revealed the molecular mechanism leading to the emergence of LMC divisions. At limb levels, the paraxial mesoderm secretes RA that induces the generation of LMC MNs (Ensini et al., 1998; Sockanathan et al., 2003; Vermot et al., 2005). Early born LMC MNs co-express ISL1/2 as well as ALDH1A2 and in turn secrete RA. This additional signal induces the down-regulation of ISL1 to the profit of the Lim homeobox 1 (LHX1) in later born LMC. Furthermore, cross-repressive interactions allow both divisions to remain mutual exclusive (Kania and Jessell, 2003). ISL1 and LHX1 also control the differential segregation of the cell body position of LMC divisions (Sockanathan and Jessell, 1998; Kania and Jessell, 2003; Rousso et al., 2008). Interestingly, matured LMCm MNs down-regulate MNX1 expression (Kania and Jessell, 2003; Rousso et al., 2008); a unique characteristic among SpMNs. Yet, the functional relevance of this distinct observation remains to be elucidated. Further information about LMC will be provided in the section dedicated to axonal targeting.

To date, 6 different motor columns have been identified in mouse the spinal cord. The SAC located in the rostral cervical segments is the only representative of the branchial category whereas the PGC in the thoracic and sacral segments is the only visceral motor column. In contrast, MMC, HMC, PMC, and LMC are somatic and innervate skeletal muscles belonging to different groups. However, to date SpMNs haven't been mapped at the single cell resolution levels (Wichterle et al., 2013). Therefore, the possibility of having uncharacterized discrete SpMN populations can't be excluded. Furthermore, SpMN diversity expands beyond the columnar organization described above. In fact, SpMNs form muscle specific groups termed pools. We will review hereafter the mechanisms driving motor pool formation.

### Specification of motor neuron pools

A remarkable event in SpMN development is the acquisition of MN pool identity, assigning to a given group a specific muscle target. The coordination between more than 50 different muscles in the typical amniotes' limb required to perform complex movements implies a precise mechanism to assign to each muscle a corresponding MN pool (Romanes, 1941; Sullivan, 1962). Previous studies have described the localization of individual MN pools according to specific targets (Landmesser, 1978; Hollyday and Jacobson, 1990; Choi and Hoover, 1996; Ryan et al., 1998) and suggest that MN pools respect a topographic organization (reviewed by Kania, 2014b). The more rostral a MN pool is positioned, the more anterior and proximal the target is located. Interestingly, MNs possess predetermined intrinsic features independent of the presence of peripheral targets that control at least partially pool specification (Phelan and Hollyday, 1990). Therefore, MN pool determination can be divided in two phases (i) purely intrinsic and (ii) extrinsically induced (Dasen, 2009).

The intrinsic molecular mechanisms of MN pool specification are not yet fully understood, however it appears to rely on the combinatorial expression of HOX proteins. Dasen et al. (2005) have performed an extensive screen of the expression of 39 *Hox* genes as well as HOX cofactors. Their results demonstrate that within a specific rostro-caudal segment, cross-repressive interactions between HOX members produce a unique combinatorial code that directs MN pool identity (Dasen et al., 2005; Lacombe et al., 2013). This identity is revealed by the activation of pool specific proteins such as the ETV1 and ETV4 (or PEA3) (Lin et al., 1998; Ladle and Frank, 2002; Livet et al., 2002), RUNX1 (Theriault et al., 2004; Dasen et al., 2005; Stifani et al., 2008; Zagami et al., 2009; Lamballe et al., 2011) and POU3F1 (Dasen et al., 2005; Rousso et al., 2008). By doing so, Dasen et al. (2005) have remarkably linked the intrinsic HOX combinatorial network to extrinsically induced factors whose expressions are dependent on a signal from the periphery (Lin et al., 1998; Haase et al., 2002) described in more detail below. However, to date the entire mapping of HOX proteins in SpMN pools remains unpublished. Furthermore, molecular effectors of pool specificity downstream of the HOX combinatorial network remain elusive.

In parallel to intra-segmental HOX combinatorial network, NKX6.1 contributes to the intrinsic mechanisms of MN pool specification (De Marco Garcia and Jessell, 2008). First, NKX6.1 is expressed in subsets of LMC MNs independently of the presence of its muscle target. Second, NKX6.1 inactivation leads to persistent muscle targeting errors. These results strongly suggest that NKX6.1 participates in controlling MN pool specificity and uncover two sequential roles of NKX6.1 in MN development. In the early phase, it takes part in the specification of progenitor domains in response to SHH gradient whereas in the late phase, it contributes to the specification of discrete MN pools. Strategically, intrinsic cues allow the development and the maturation of MNs independently of their location. This approach provides plasticity and tolerance to adapt to changes in the peripheral environment.

Unlike NKX6.1 and the HOX combinatorial intra-segmental network described above, extrinsically induced players are expressed in developing MNs upon reception of a specific signal. This mechanism can be considered as a checkpoint ensuring further developmental refinements only after the completion of prerequisite steps. What are the extrinsic signals allowing further MN differentiation? So far, only one factor has been unambiguously identified. Namely, the glial cell derived neurotrophic factor (GDNF) is secreted by both *Cutaneous maximus* (CM) and *Latissimus dorsi* (LD) muscles and induces the expression of ETV4 in the corresponding MN pools (Lin et al., 1998; Haase et al., 2002; Livet et al., 2002). The analysis of ETV4 mutant animals revealed that even though some aspects of MN development are pre-established by intrinsic cues, later signals are further required for the maintenance of MN pool characteristics such as cell body position, axonal arborization and dendritic patterning ensuring the establishment of correct input connections (Ladle and Frank, 2002; Livet et al., 2002; Vrieseling and Arber, 2006). Additionally, after the initial expression of ETV4 induced by GDNF, CM MNs recruits adjacent MNs and induce in a non-cell autonomous manner the expression of ETV4 (Helmbacher et al., 2003). Therefore, one of the strategy initial differentiation followed by the recruitment *in situ* of neighboring MNs.

Together these results illustrate the coordination between intrinsic and extrinsically-induced cues. While the first group allows MN development independently of the environment, the second ensures the completion of essential steps. Together these mechanisms create a flexible process allowing MNs to adapt to environment variability. By definition, MN pool specification is intimately linked to axonal targeting. Intensive works have identified various molecules involves in SpMN axonal targeting. We will dedicate the next section to the review the known molecular mechanisms controlling SpMN axonal targeting.

### Motor neuron axonal targeting

**Figure 9:**
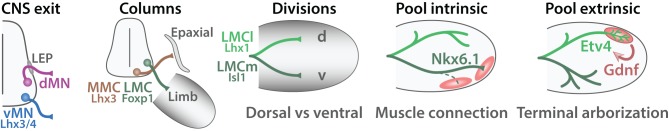
**Steps of MN axonal targeting**. Schematic summarizing the steps of MN axonal targeting (adapted from Dasen and Jessell, 2009). The first step termed “CNS exit” reflect the choice of developing MNs to exit the CNS via the ventral root (vMNs, blue) or through the lateral exit point (LEP) (dMNs, red). The second choice labeled “Columns” return to the motor column: MMC MNs (brown) target to epaxial musculature whereas LMC MNs (green) project to the limb. The third step named “Divisions” refers to the choice made by the medial and lateral divisions of the LMC. LMCl MNs (light green) invade the dorsal part of the limb (d) whereas LMCm (dark green) MNs target to the ventral region (v). The fourth step termed “Pool intrinsic” refers to the selection of a specific muscle target (red) and is controlled by intrinsic cues. The last step named “Pool extrinsic” illustrates the induction of specific protein expression upon a signal from the muscle target, which coordinates the terminal arborization of MN axons. Proteins involved in each step are indicated.

Axonal targeting is a critical process of MN development. MN axons emerge within the CNS and transit through different tissues to reach and connect to their specific muscle target in the periphery. Axonal targeting not only provides to MNs their unique anatomical characteristic and therefore their irreplaceable function but also ensures their persistence through the action of trophic signals. In order to complete such critical process, MNs combine several mechanisms in a stepwise manner (Figure 9). Several “checkpoints” are established along the axonal route, each one requiring a choice to orientate toward a particular direction. While the initial steps rely on intrinsic mechanisms, the late aspects of MN axonal targeting rely on signals received at the growth cone, and inducing molecular and anatomical modifications.

**Figure 10:**
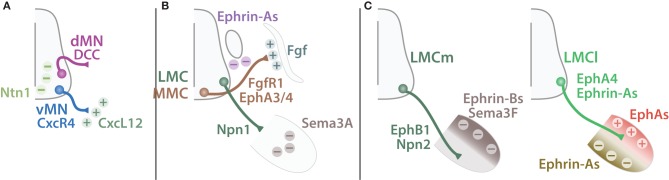
**Guiding cues of SpMN axonal targeting**. Schematic summarizing guiding cues important for MN axonal targeting. **(A)** Ventral exiting MNs (vMN, blue) express CxcR4 and are attracted (plus signs) by CxcL12 expressed by the mesenchyme (dark green). Dorsal MNs (dMN, purple) express DCC and are repelled (minus sign) away from the midline expressing Ntn1 (light green). **(B)** MMC MNs (brown) expressing both FgfR1 and EphA3/4 are attracted by Fgf secreted by the dermomyotome but repelled by Ephrin-As expressed by the dorsal root ganglion. LMC MNs (green) target to the limb and pause before further growth. This pause is mediated by Npn1-Sema3A repellent signaling expressed by LMC MNs and the limb respectively. **(C)** LMCm MN (dark green) axons express EphB1 and Npn2 and are constrained into the ventral limb by Sema3F and Ephrin-Bs expressed by the dorsal limb mesenchyme (dark brown). Conversely, LMCl MN (light green) axons express Ephrin-As and EphA4 and are restricted to the dorsal part of the limb by a combination of Ephrin-As repulsive signal from the ventral limb mesenchyme (light brown) and EphAs (red) attractive signal from the dorsal part of the limb.

The very first choice SpMN axons make occurs within the spinal cord (termed “CNS exit” in Figure 9). vMNs leave the CNS via the ventral root whereas dMNs exit more dorsally via the LEP. This decision is at least partially controlled by LHX3 and 4 (Sharma et al., 1998; Bravo-Ambrosio and Kaprielian, 2011). Yet, LHX3/4 are transcription factors and therefore are unlikely the effectors of this axonal targeting decision made at the growth cone. Instead, the chemokine (C-X-C motif) receptor 4 (CXCR4) is expressed by vMN axons and its ligand CXCL12 localizes in the ventral mesenchyme surrounding the spinal cord. This molecular signal attracts vMN axons toward the ventral root (Lieberam et al., 2005). Conversely, dMNs express the netrin receptor deleted in colorectal carcinoma (DCC) and are repelled away from the midline expressing netrin 1 (NTN1) (Dillon et al., 2005) (Figure 10A). The complete molecular mechanisms allowing dMNs to escape the classical ventral root exit are yet to be characterized. As dMNs are absent outside of the cervical regions, novel molecules involved in SAC MNs axonal targeting could presumably be restricted to the first cervical segments. Unbiased differential screenings of genes downstream of transcription factors exclusive of dMNs (PHOX2B) or vMNs (LHX3/4) may identify new effector molecules involved in their divergence.

The second step in MN axonal growth consists in selecting the orientation toward their forthcoming muscle target (termed “Columns” in Figure 9). Schematically, growing vMN axons can adopt three directions: (i) dorsal, toward the axial musculature (MMC), (ii) lateral, invading the limb (LMC) or (iii) ventral, toward the sympathetic chain or to the body wall musculature (PGC and HMC, respectively). This schematic intentionally omits PMC targeting for simplicity. These decisions are comprised within the identity of a particular motor column and therefore considered as intrinsic. Presumably, the unique combinatorial expression of transcription factors controls downstream effectors and modulators of axonal growth. Although the molecular mechanisms remain largely unknown, MMC MNs express the fibroblast growth factor receptor 1 (FGFR1) and are attracted by the dermomyotome secreting FGF (Shirasaki et al., 2006; Soundararajan et al., 2010). Additionally, MMC axons expressing the Eph receptor A3 and 4 (EPHA3 and 4) are constrained by repellent contact with sensory DRG neurons expressing ephrin-As (EFNA1) (Gallarda et al., 2008). Together these mechanisms lead MMC axons to bypass the DRG and target to the axial musculature (Figure 10B). The molecules leading LMC axons to initially target the limb are unknown, however Huber et al. (2005) revealed the role of Semaphorin-Neuropillin in controlling the timing and the fasciculation of LMC axons. Neuropilin 1 (NRP1) expressed by LMC axons mediates the repulsion from the limb mesenchyme expressing semaphoring 3A (SEMA3A). Inactivation of SEMA3A-NRP1 signaling results in a premature invasion of the limb bud. Interestingly, NRP1 is expressed by both MN and SN axons and contributes to MN axon fasciculation along the sensory axons (Huettl et al., 2011). This example illustrates the use of a single molecule to synchronize sensory and motor development (Wang et al., 2011; Fukuhara et al., 2013). Such strategy ensures the formation of a coherent and functional circuitry. Lastly, PGC and HMC axons specifically turn ventrally toward the sympathetic chain and the body wall musculature, respectively. To date the mechanisms of such decision remain unidentified.

The lateral and medial divisions of the LMC have provided a powerful framework to study MN axonal decisions. After entering the base of the limb LMC axons pause before targeting toward the dorsal or the ventral parts of the limb (Tosney and Landmesser, 1985a; Wang and Scott, 2000). LMCm MNs express ISL1 and target to the ventral part of the limb whereas LMCl MNs express LHX1 and connect to the dorsal part of the limb (termed “Division” in Figure 9). Interestingly, LHX1 inactivation does not perturb LMCl formation but instead impairs the dorsal/ventral axonal projection specificity (Kania et al., 2000). Reciprocally, the LIM homeobox transcription factor 1 beta (LMX1B) expressed in a decreasing dorsal to ventral gradient in the limb mesenchyme is also important for LMC divisions axonal targeting (Kania et al., 2000). The molecular mechanisms of LMC axonal targeting rely prominently on Ephrin-Eph signaling and have been the source of recent exciting discoveries summarized by Bonanomi and Pfaff (2010) and reviewed in depth by Kao et al. (2012). In brief, LMCl MNs express LHX1 that induces the expression of EPHA4. LMCl axons are repelled away from the ventral limb mesenchyme expressing EFNAs (Helmbacher et al., 2000; Kania and Jessell, 2003). Similarly, LMCm MNs express EPHB1 are repulsed from the dorsal limb mesenchyme expressing EFNBs (Luria et al., 2008). Therefore, cross-repulsive Ephrin-Eph signaling mediates the correct segregation of LMCl and LMCm (Figure 10C). However, additional mechanisms contribute as well to LMC MNs axonal targeting. For example, GDNF and GDNF family receptor alpha 1 (GFRA1) cooperate with EFNAs-EPHAs signaling to control LMC MN dorso-ventral choice (Kramer et al., 2006). More recently, new discoveries have enriched Ephrin-Eph signaling with additional levels of complexity. Trans forward and reverse signaling (Dudanova et al., 2012) as well as interaction in *cis* (Kao and Kania, 2011) regulate LMC MN axonal targeting. Furthermore, the tyrosine kinase receptor Ret proto-oncogene (RET) acts co-receptor for both GDNF and ephrin-As modulating their response and thus adding another layer of complexity in LMC MN axonal targeting (Bonanomi et al., 2012). Together these results demonstrate that LMC targeting is complex and tightly regulated. Further experiments will permit a better understanding of this multifaceted process.

After making their initial decisions MN axons need to select their specific muscle target. This step is closely related to the formation of MN pools discussed above. MNs are programmed to recognize their muscle target (Lance-Jones and Landmesser, 1980). Intrinsic cues are expressed in a pool specific manner to direct MN axons toward their specific muscle target (termed “Pool Intrinsic” in Figure 9). NKX6 (De Marco Garcia and Jessell, 2008) as well as the HOX combinatorial network (Dasen et al., 2005) have been proposed as intrinsic regulators of muscle target selection. Presumably, other molecules, yet to characterize, play a role in the establishment of specific connections between a MN pool and its respective muscle target. Among them, the downstream molecular effectors that regulate axonal path finding remain to be identified.

Finally, after reaching their appropriate muscle, MN axons need to form functional connections with their target. Interestingly, studies focusing on the CM and LD muscles have revealed that this process is initiated upon receiving a signal from the peripheral target and therefore is considered as an extrinsic event (termed “Pool extrinsic” in Figure 9). MN pools innervating these two muscles are characterized by the expression of ETV4 (Ladle and Frank, 2002). It has been remarkably shown that the initial expression of ETV4 is induced by GDNF expressed by the CM and LD muscles (Haase et al., 2002; Helmbacher et al., 2003). In turn, ETV4 is responsible for inducing the terminal axonal arborization (Livet et al., 2002) as well as the dendritic refinement of these specific MN pools (Vrieseling and Arber, 2006). The molecular effectors of terminal arborization are still unknown; however, downstream targets of GDFN and/or ETV4 signaling could be good candidates for further investigation. Recently, Audouard et al. (2012) have identified for the first time a transcriptional regulator of neuromuscular junction formation. The analysis of ONECUT1 inactivated animals demonstrates a peculiar hind limb locomotion pattern resulting from impairments in neuromuscular junction formation. These findings open new opportunities to further characterize downstream molecular effectors important for the formation of functional connections between MNs and their respective muscle targets.

### Period of natural cell death

MNs are generated in excess and then progressively decrease in number during a natural cell death period (Oppenheim, 1991). This process ensures the generation of the appropriate number of MNs and guarantees the elimination of aberrant cells. This strategy can also result from the requirement of a temporary function; for example, certain MNs may initially be generated to ensure a particular developmental function and are subsequently eliminated. Regardless of the reasons, natural MN death leads to the removal of around 40% of the initially generated MNs (Hamburger, 1975). This loss can be comprehensively divided into two phases (Yaginuma et al., 1996). The early phase is independent of any peripheral signal and likely reflects a negative selection of unsuitable MNs. The subsequent phase has been described more intensively and is dependent on survival signals from the periphery and thus reflects the refinement of mature MN innervations. Temporally, natural MN cell death in mice starts progressively from embryological day (E) 11.5 in most rostral segments and spreads gradually to the caudal levels with a peak occurring at E14 (Yamamoto and Henderson, 1999). The absence of MN cell death postnatally suggests a necessity to reach completion of MN development before birth (Oppenheim, 1986). Numerous molecules have been involved in MN survival signaling. The initial discoveries of the nerve growth factor (NGF), neurotrophins (NTFs) and brain derived neurotrophic factor (BDNF) (Snider, 1994) led to the characterization of additional molecules involved in neuronal survival, including cytokines (ciliary neurotrophic factor CNTF, leukemia inhibitory factor LIF) (Dechiara et al., 1995; Li et al., 1995), the TGFB family (GDNF, neurturin NRTN, persephin PSPN) (Henderson et al., 1994; Poulsen et al., 1994; Oppenheim et al., 1995, 2000), the hepatocyte growth factor (HGF) as well as FGF1, 2 and 5 (Henderson, 1996; Oppenheim, 1996).

Interestingly, in parallel of the general survival mechanisms introduced above, results suggest the existence of pool specific survival signals (Gould and Oppenheim, 2004). Gu and Kania (2010) undertook the profiling of survival receptors expression in lumbar LMC MN pools as well as survival molecules in the corresponding limb muscles. Although their results did not reveal a general mechanism linking MN pool specific survival and combination of trophic factors expressed in the muscles, they emphasized the complexity of MN survival. Indeed, the authors discuss several indications supporting a plausible convergence between the mechanisms controlling axon guidance and MN survival into a unified and coherent process.

Since this article does not primarily focus on MN cell death and selective survival, the following reviews are recommended to provide a detailed description of this complex and indispensable process (Oppenheim, 1991; Hamburger, 1992; Henderson, 1996; Pettmann and Henderson, 1998; Gould and Enomoto, 2009).

### Specification of motor neuron subtypes within a pool

The diversity of SpMNs is not limited to the specification of MN pools but also impinges on muscle fiber structural and functional diversity. Despite the detailed columnar classification of SpMNs, little is known about the mechanisms causing a specific MN to recognize and connect to a unique fiber type within its individual muscle target. We will describe recent studies that shed light on the mechanisms controlling alpha and gamma MN differentiation as well as between fast and slow alpha MNs.

#### Alpha vs. gamma MNs

The divergence between alpha and gamma MNs is poorly characterized (Eccles et al., 1960; Bryan et al., 1972; Westbury, 1982). Evidence from several studies suggests that alpha and gamma MN identities are fated early during embryonic stages. For example, inactivation of the programmed cell death in MNs leads to an increased number of MNs with gamma characteristics (Buss et al., 2006). This result implies that alpha and gamma MN are differentiated prior to axon outgrowth and trophic support requirement. During the first weeks after birth, alpha and gamma MNs can be molecularly identified by the differential expression of the RNA binding protein, fox-1 homolog 3 (RBFOX3 or NeuN), the estrogen-related receptor gamma (ESRRG) (Friese et al., 2009), the GDNF family receptor alpha 1 (GFRA1) (Shneider et al., 2009), the serotonin receptor 1D (HTR1D) (Enjin et al., 2010) as well as the ATPase, Na+/K+ transporting, alpha 1 (ATP1A1) (Edwards et al., 2013). Alpha MNs maintain high levels of RBFOX3 expression after birth whereas gamma MNs up-regulate ESRRG and GFRA1 and simultaneously down-regulate RBFOX3. These markers are segregated only at post-natal stages and are therefore unlikely participate in the early phase of alpha and gamma MN divergence. A recent study identified the first embryological marker of gamma MNs (Ashrafi et al., 2012). Namely, WNT7A is selectively expressed in gamma MNs at late embryological stages. The authors also revealed that its expression is dependent on a muscle spindle-derived signal that is not GDNF, previously characterized as required for their survival (Gould et al., 2008; Shneider et al., 2009). These results open new perspectives to further characterize the molecular mechanisms controlling alpha *vs.* gamma MN divergence.

#### Fast vs. slow MNs

Alpha MNs can be classified according to the type of extrafusal fiber they innervate (FF, FFR, SFR). MNs are intrinsically competent to recognize and connect to either fast or slow muscle fibers (Rafuse et al., 1996; Landmesser, 2001). Studies have proposed that the synaptic vesicle glycoprotein 2a (SV2A) (Chakkalakal et al., 2010) as well as the estrogen-related receptor beta (ESRRB) (Enjin et al., 2010) are restricted to slow MNs soon after birth. Conversely, the calcitonin-related polypeptide alpha (CALCA) and the chondrolectin (CHODL) are restricted to fast MNs (Enjin et al., 2010). More recently, Muller et al. (2014) elegantly identified the non-canonical Notch ligand delta-like homolog 1 (DLK1) as a regulator necessary and sufficient to promote fast MN phenotype. These results identify for the first time a molecular regulator of the fast vs. slow MN divergence. This initial breakthrough will indubitably facilitate further identification of the mechanisms of fiber-type-specific alpha MN differentiation.

In summary, SpMN diversity expends beyond the formation of MN pools. In fact, SpMN identity impregnates into muscle fiber types characteristics. Recent findings lead to the identification of key players as well as molecular markers of MN subtype populations. These discoveries open new avenue for further characterization.

### Conclusion and perspectives on the generation of spinal motor neurons

SpMNs are unique and irreplaceable neuronal cells connecting the CNS to targets in the periphery. While visceral SpMNs of the thoracic and sacral regions control autonomic functions, somatic SpMNs regulate movements by controlling the contraction of individual muscles. These crucial roles lead to inexorable impairments when affected by diseases. Thus, intensive research has focused on understanding MN biology and diseases.

Over the years, studies have accumulated data and revealed mechanisms driving MN properties and behaviors. Remarkably, the diversity of SpMNs mirrors the variety of targets they innervate but also impinges within individual muscle fiber types. This exceptional diversity is acquired progressively during development and has been reviewed here. The ventralization of the neural tube has been described as a consequence of surrounding molecules expressed in a gradient fashion and inducing in a concentration dependent manner the expression of sets of homeodomain proteins leading the emergence of exclusive progenitor domains. All SpMNs arise from the pMN domain from which SpMN precursors exit the cell cycle and migrate away from the neuroepithelium while acquiring post-mitotic MN features. Concomitantly, patterning molecules along the rostro-caudal axis induce in a concentration-dependant manner the expression of several transcription factors notably members of the HOX family. In turn, these proteins define exclusive rostro-caudal segments (brachial, thoracic, lumbar). Subsequently, while SpMNs strengthen their motor identity, they segregate into anatomical columns termed motor columns. Combinations of LIM homeodomain proteins provide a unique molecular profile for each motor column. In parallel, the LIM code induces the initial steps of a crucial process: MN axonal targeting. SpMN axonal targeting and further differentiation occurs in a step-wise manner. Checkpoints are established along the route to ensure the completion of critical steps. Furthermore, these checkpoints are informative and instruct developing SpMNs of the environment at the growth cone. The SpMN target can be seen as the last checkpoint of the chain. Upon reaching their final destination, SpMNs are required to complete their differentiation process and form functional connection with their target. SpMN identity echoes muscle fiber type properties. Finally, as a mechanism controlling the integrity of SpMN development, naturally programmed cell death induces the elimination of inadequate MNs and ensure the formation of a coherent circuitry.

Although, the overall strategy as well as the intrinsic transcription factors governing the generation of SpMN diversity have been, at least partially characterized and summarized here, our review emphasizes the poor knowledge about the downstream molecular effectors of MN development. In fact, the more differentiated SpMNs become the more fragmentary our understanding is. This is particularly important in regard to prospective MN regeneration therapies for which understanding MN general identity will not be sufficient. Instead, tweaking subtype-specific effector molecules may be a powerful strategy to regenerate functional MNs in fully developed adults. The identification of additional effectors can be achieved in two ways: (i) oriented investigation of downstream targets of known intrinsic regulators such as LIM and/or HOX proteins for example and (ii) unbiased screenings combining, viral retrograde tracing (Stepien et al., 2010; Tripodi et al., 2011), laser capture micro-dissection (Bandyopadhyay et al., 2014) and RNA sequencing (Enjin et al., 2010). Such approaches would indubitably unveil new regulators and effectors of SpMN subtype specification. Furthermore, several studies have already shed light on the role of non-coding micro RNAs (miRNAs) in MN development (Cao et al., 2007; Visvanathan et al., 2007; Otaegi et al., 2011a,b). For example, Chen and Wichterle (2012) demonstrate that the inactivation of the Endoribonuclease Dicer (DICER1), an important player of double strand RNA post-transcription gene silencing, perturbs the formation of PGC and LMC MNs. Similarly, OLIG2 repression initiated at the p2-pMN border relies on mir-17–3 p miRNA-mediated silencing of *Olig2* mRNA (Chen et al., 2011a). The implication of non-coding miRNAs is likely more complex and numerous findings will likely arise from this recent and mostly unexplored field of research. The unbiased screenings mentioned above could identify novel regulatory mechanisms of SpMN diversity involving non-coding RNAs.

SpMNs are anatomically well organized. This morphological arrangement correlates with the position of their respective target in the periphery as reviewed by Kania (2014b). Thus, SpMN settling position and axonal targeting must be somehow molecularly connected. An ingenious strategy to further understand the mechanisms driving SpMN specification consists in uncoupling MN differentiation processes such as column formation, cell body positioning, and axonal targeting. One naturally occurring opportunity to study MN differentiation processes independently from one another could lie on the analysis of *rhomboideus* MN pool. These neurons constitute, in fact, the only known exception to the MN columnar organization described earlier. Although innervating an axial muscle, this MN pool is located in the lateral component of the ventral horn at caudal brachial segments; a position typical of LMC MNs (Straznicky and Tay, 1983; Hollyday and Jacobson, 1990; Tsuchida et al., 1994; Rousso et al., 2008). Therefore, molecular profiling of this particular MN pool may be interesting to identify new effectors and regulators of SpMN organization.

Finally, this review deliberately focused on SpMN development from a motor perspective. However, SpMNs are “only” one constituent of a larger coherent circuitry. Complex movements require the control of individual muscles in a collaborating manner. This coordination relies on a highly organized circuitry between SNs, association neurons, and SpMNs as reviewed by Ladle et al. (2007). In a perspective of regeneration therapies, SpMNs with the correct identity should insert in a pre-existing neuronal circuitry. Such possibility infers that (i) regenerated SpMNs settle at their appropriate location, (ii) that SpMNs' inputs are plastic to form new functional connections and (iii) that regenerated SpMNs project to their appropriate target across a fully developed living organism. These are the challenges the scientific MN community will have to resolve in the coming future.

## Author contributions

Nicolas Stifani reviewed the relevant literature, wrote the manuscript and generated the figures.

### Conflict of interest statement

The author declares that the research was conducted in the absence of any commercial or financial relationships that could be construed as a potential conflict of interest.
